# Global, regional, and national burdens of bladder cancer in 2017: estimates from the 2017 global burden of disease study

**DOI:** 10.1186/s12889-020-09835-7

**Published:** 2020-11-11

**Authors:** Hairong He, Hongjun Xie, Yule Chen, Chengzhuo Li, Didi Han, Fengshuo Xu, Jun Lyu

**Affiliations:** 1 Clinical Research Center, The First Affiliated Hospital of Xi’an Jiaotong University, Xi’an, 710061 Shaanxi China; 2 School of Public Health, Xi’an Jiaotong University Health Science Center, Xi’an, Shaanxi China; 3 Department of Urology, The First Affiliated Hospital of Xi’an Jiaotong University, Xi’an, Shaanxi China; 4 Department of Clinical Research, The First Affiliated Hospital of Jinan University, Guangzhou, China

**Keywords:** Bladder cancer, GBD 2017, Prevalence, Mortality

## Abstract

**Background:**

The aim of this study is to describe the prevalence and mortality of bladder cancer (BCa) using data obtained in the Global Burden of Disease study performed in 2017 (GBD 2017).

**Methods:**

Data on BCa for 2017, including prevalence, mortality, and disability-adjusted life years (DALYs), were obtained from GBD 2017 at the global, regional, and national levels. We also analyzed the association of BCa burden with the country development level.

**Results:**

There were 2.63 million BCa cases estimated from the GBD 2017 data, with 200,000 persons dying of BCa, resulting in 3.60 million DALYs in 2017. The age-standardized prevalence (ASP) of BCa was 32.91/100,000 persons, and age-standardized death rate (ASDR) was 2.57/100,000 persons. The ASP and ASDR of BCa were higher in males than in females, and higher in people older than 60 years. The ASP and ASDR of BCa were higher in Western Europe and Central Europe than in South Asia, Andean Latin America, and Central Latin America, and higher in countries with a higher sociodemographic index (SDI). Correlation analysis identified that the ASP and ASDR of BCa were positively correlated with the country SDI (*P* < 0.0001 and *ρ* = 0.68 for ASP, and *P* = 0.0048 and *ρ* = 0.20 for ASDR). In addition, 33.72% deaths and 36.80% DALYs caused by BCa could be attributed to smoking globally.

**Conclusion:**

The prevalence and mortality of BCa were very high in 2017, especially in high-SDI countries. Smoking-cessation strategies should be strengthened to control the burden associated with BCa.

**Supplementary Information:**

The online version contains supplementary material available at 10.1186/s12889-020-09835-7.

## Background

Bladder cancer (BCa) is one of the most-common urological malignancies, and is among the leading causes of cancer deaths worldwide. Approximately 3.0% of all new cancer diagnoses and 2.1% of all cancer deaths are due to BCa [1]. The high incidence, prevalence, mortality, and recurrence rate of BCa indicate that it remains an unsolved clinical and social problem. Two-thirds of patients present with non-muscle-invasive BCa. Although current therapies can achieve a good prognosis, approximately 40% of these patients will progress to muscle-invasive disease after 5 years [2–4]. Moreover, the 5-year survival rate of muscle-invasive BCa is only 60% [5].

Richters A, et al. systematically described the epidemiological characteristics of BCa. They concluded the burden of BCa exhibits obvious regional, sex, and age variations [6]. Specifically, the incidence rate of BCa is about threefold higher in Europe and North America than that in South-East Asia, Latin America and Northern Africa [6]. Worldwide, the lifetime risk of getting BCa is 1.1% in males and 0.27% in females [6]. Most patients are diagnosed with BCa when they are aged at least 60 years (SEER data) [6]. Understanding how the disease burden of BCa varies between countries, sex, and age is necessary for policymakers to efficiently allocate the limited available resources.

The Global Burden of Disease study performed in 2017 (GBD 2017) estimated the burdens of diseases, injuries, and risk factors at the global, regional, and national levels, and how they varied with sex and age [7]. The data sources of GBD 2017 was based on published literature, surveillance data, survey data, hospital and clinical data, and other types of data, which was produced by 3676 collaborators from 146 countries and territories. Then the GBD study applied different methods (the standard approach was the Bayesian metaregression tool DisMod-MR 2.1.) to utilize raw data and to measure specific epidemiological patterns of disease burden [7]. Presentation of GBD 2017 results is based on a geographical hierarchy that includes 195 countries and territories grouped into 21 regions and seven GBD super-regions. In short, GBD 2017 provides an opportunity to comprehensively characterize the distribution of BCa.

Ebrahimi H, et al. [8] and Dy GW, et al. [9] described the disease burden of bladder cancer using GBD 2016 and GBD 2013 data, respectively. However, the characteristics of the global BCa burden in 2017 are still unknown. The prevalence status of BCa is also unknown because the two published articles used incidence and mortality as their epidemiological indicators. Based on these two points, this study was designed to describe the prevalence, mortality, and disability-adjusted life years (DALYs) of BCa in 2017 according to sex, age, sociodemographic index (SDI), region, and country. We also attempted to identify the burden of BCa attributable to risk factors based on GBD 2017 data.

## Method

The results obtained in GBD 2017 are now available via an interactive data-downloading tool using the GHDx (Global Health Data exchange) software (http://www.healthdata.org/gbd/). Users can retrieve data on different aspects of a disease by choosing the appropriate selection boxes [10]. In the present study we collected information on the prevalence, mortality, and DALYs of BCa in 2017 according to sex, 21 regions, and 195 countries or territories. We also analyzed the prevalence, mortality, and DALYs of BCa according to the 15 age groups used in GBD 2017: 14 5-year periods from 10 to 79 years, plus > 79 years. Additionally, the GBD study quantifies the burden of several causes and impairments attributable to 84 environmental and occupational, metabolic, and behavioral risk factors. So the attributable risk factors for the BCa burden were also analyzed in this study.

The age-standardized rates (ASRs) in GBD 2017 were obtained for the global age-standardized population [7]. The ASR was calculated by summing up the products of the age-specific rates (*a*_*i*_, where *i* is the *i*th age class) and the number of persons (or weight *w*_*i*_) in age subgroup *i* of the selected reference standard population, then dividing by the sum of the standard population weights: ASR $=\frac{\sum \limits_{i=1}^A{a}_i{w}_i}{\sum \limits_{i=1}^A{w}_i}\times \mathrm{100,000}$. Namely, the age-standardized prevalence (ASP) corresponds to the number of cases per 100,000 persons, the age-standardized death rate (ASDR) corresponds to the number of deaths per 100,000 persons, and the ASR of DALYs corresponds to the years lived with disability and years of life lost per 100,000 persons after age standardization.

The SDI is a composite indicator of development status that is strongly correlated with health outcomes, and is calculated as the geometric mean of 0 to 1 indices of the total fertility rate among those younger than 25 years, the mean education level among those aged at least 15 years, and the lag distributed income per capita. GBD 2017 divides both countries and regions into the following five SDI quintiles: high, high-middle, middle, low-middle, and low. We compared the burden of BCa among these five SDI quintiles. In addition, we explored the factors influencing ASR by evaluating the associations of ASR with the SDI in 2017 at the country level using Pearson correlation analysis, with *P* < 0.05 considered indicative of statistical significance.

All of the figures were made through the R package (version 3.5.1; https://www.r-project.org/) based on data obtained in GBD 2017.

## Results

### Global burden of BCa

As indicated in Table 1, the prevalence, mortality, and DALYs of BCa were higher in males than in females. It was estimated from GBD 2017 data that there were 2.63 million (95% confidence interval [CI] = 2.57–2.72 million) BCa cases, involving 2.03 million (95% CI = 1.96–2.11 million) males and 0.60 million (95% CI = 0.58–0.62 million) females. There were 200,000 (95% CI = 190,000–210,000) deaths from BCa, including 140,000 (95% CI = 140,000–150,000) males. BCa resulted in 3.60 million (95% CI = 3.48–3.77 million) DALYs, comprising 2.71 million (95% CI = 2.60–2.87 million) in males and 0.89 million (95% CI = 0.85–0.92 million) in females. The ASP of BCa was 32.91/100,000 persons (95% CI = 32.09–33.95/100,000 persons), the ASDR of BCa was 2.57/100,000 persons (95% CI = 2.51–2.69/100,000 persons), and the ASR of DALYs of BCa was 45.27/100,000 persons (95% CI = 43.73–47.42/100,000 persons). Similarly, the ASP, ASDR, and ASR of DALYs of BCa were higher in males than in females.

**Table 1:** The prevalent cases, death number and DALYs and their age-standardized rate of bladder cancer in 2017

Location	Prevalent cases (No. × 10^**4**^)	ASPR (per 100,000 persons)	Death number (No. × 10^**3**^)	ASDR	DALYs number (No. × 10^**4**^)	ASR of DALYs (per 100,000 persons)
**Global**	263.24 (256.69–271.72)	32.91 (32.09–33.95)	196.55 (191.55–205.84)	2.57 (2.51–2.69)	359.72 (347.54–376.64)	45.26 (43.73–47.42)
**Sex**						
Male	202.92 (196.28–211.47)	54.64 (52.87–56.91)	144.71 (139.56–153.99)	4.39 (4.24–4.67)	270.81 (259.57–287.45)	74.23 (71.16–78.66)
Female	60.31 (58.35–62.05)	14.19 (13.73–14.6)	51.84 (50.31–53.33)	1.2 (1.17–1.24)	88.9 (85.42–92.4)	20.89 (20.07–21.71)
**Socio-demographic index**						
High SDI	133.29 (129.02–137.59)	62.51 (60.51–64.54)	85.78 (83.36–88.16)	3.44 (3.34–3.53)	132.45 (127.53–137.79)	59.66 (57.4–62.14)
High-middle SDI	60.15 (58.02–63.7)	33.16 (32.-35.09)	42.98 (41.61–45.44)	2.52 (2.44–2.66)	83.23 (80.03–87.57)	46.5 (44.74–48.91)
Low SDI	7.13 (6.5–8.41)	10.16 (09.28–12.)	10.87 (9.96–12.67)	1.86 (1.7–2.19)	23.83 (21.78–27.57)	34.55 (31.61–40.12)
Low-middle SDI	22.16 (20.02–24.64)	17.78 (16.29–19.7)	22.83 (21.38–25.13)	2.2 (2.06–2.45)	51.97 (47.97–56.86)	43.53 (40.41–47.58)
Middle SDI	39.62 (37.73–43.67)	17.71 (16.88–19.54)	33.42 (31.81–37.15)	1.69 (1.61–1.88)	67.02 (63.16–73.49)	30.81 (29.05–33.88)
**Region**						
Andean Latin America	0.67 (0.6–0.76)	12.38 (11.-13.95)	0.74 (0.66–0.84)	1.4 (1.26–1.6)	1.32 (1.17–1.49)	24.58 (21.9–27.87)
Australasia	2.34 (2.11–2.59)	49.35 (44.39–54.57)	1.51 (1.37–1.65)	2.81 (2.56–3.09)	2.25 (2.03–2.48)	45.91 (41.38–50.66)
Caribbean	1.45 (1.34–1.59)	28.57 (26.34–31.33)	1.33 (1.24–1.44)	2.62 (2.43–2.84)	2.41 (2.22–2.62)	47.38 (43.72–51.63)
Central Asia	1.8 (1.7–1.89)	22.74 (21.63–23.83)	1.45 (1.37–1.53)	2.2 (2.09–2.34)	3.32 (3.14–3.51)	44.56 (42.15–47.1)
Central Europe	12.79 (12.3–13.27)	61.55 (59.18–63.94)	10.12 (9.76–10.48)	4.5 (4.34–4.67)	18.76 (17.97–19.59)	87.47 (83.82–91.32)
Central Latin America	3.47 (3.32–3.64)	14.76 (14.12–15.47)	3 (2.88–3.13)	1.36 (1.3–1.42)	5.79 (5.53–6.06)	25.04 (23.96–26.21)
Central Sub-Saharan Africa	0.69 (0.55–0.9)	14.03 (11.27–19.)	1.08 (0.86–1.45)	2.85 (2.2–4)	2.51 (2.01–3.29)	52.32 (41.97–70.15)
East Asia	45.05 (42.61–50.79)	21.93 (20.77–24.6)	32.75 (31.03–37.53)	1.77 (1.68–2.03)	62.14 (58.52–71.54)	30.94 (29.18–35.37)
Eastern Europe	14.07 (13.51–14.59)	41.88 (40.22–43.41)	10.53 (10.2–10.84)	2.97 (2.88–3.06)	21.37 (20.57–22.22)	61.98 (59.6–64.54)
Eastern Sub-Saharan Africa	2.00 (1.73–2.23)	12.79 (11.09–14.34)	3.17 (2.73–3.56)	2.47 (2.14–2.76)	7.17 (6.18–8.05)	46.94 (40.34–52.71)
High-income Asia Pacific	16.47 (15.55–17.34)	38.79 (36.47–41.04)	11.15 (10.76–11.57)	2.04 (1.96–2.12)	14.92 (14.15–15.79)	32.64 (30.74–34.76)
High-income North America	36.79 (35.71–37.98)	61.33 (59.46–63.31)	21.94 (21.35–22.68)	3.34 (3.25–3.45)	36.13 (34.78–37.51)	59.14 (56.88–61.48)
North Africa and Middle East	19.6 (17.78–22.28)	43.67 (39.98–49.78)	12.58 (11.56–15.21)	3.35 (3.08–4.17)	29.45 (26.75–33.62)	68.69 (62.87–80.26)
Oceania	0.11 (0.09–0.13)	14.91 (12.43–16.98)	0.1 (0.08–0.12)	1.97 (1.59–2.24)	0.28 (0.23–0.33)	40.88 (33.37–46.71)
South Asia	14.34 (13.36–16.14)	10.57 (9.86–11.88)	18.87 (17.53–21.25)	1.67 (1.55–1.89)	40.22 (37.35–44.86)	30.35 (28.19–34.03)
Southeast Asia	11.43 (09.96–12.55)	18.82 (16.42–20.64)	10.13 (8.54–11.3)	2 (1.69–2.23)	21.41 (18–23.93)	37.1 (31.23–41.46)
Southern Latin America	2.94 (2.7–3.21)	36.18 (33.2–39.53)	2.51 (2.32–2.72)	2.97 (2.73–3.22)	4.42 (4.04–4.87)	53.78 (49.03–59.16)
Southern Sub-Saharan Africa	1.17 (1.02–1.26)	20.32 (17.62–21.86)	1.36 (1.2–1.47)	2.74 (2.44–2.95)	3.03 (2.68–3.31)	54.04 (47.59–58.65)
Tropical Latin America	5.25 (5.11–5.42)	22.42 (21.78–23.12)	4.95 (4.81–5.1)	2.24 (2.18–2.31)	9.31 (9–9.62)	40.37 (39.06–41.74)
Western Europe	68.44 (65.08–71.88)	80.17 (76.12–84.19)	43.84 (42.02–45.62)	4.21 (4.02–4.38)	65.9 (62.57–69.3)	73.07 (69.24–77.13)
Western Sub-Saharan Africa	2.37 (2.00–2.74)	12.93 (10.9–14.91)	3.46 (2.91–3.97)	2.34 (1.97–2.69)	7.6 (6.37–8.76)	43.09 (36.16–49.46)

*ASPR* age-standardized prevalence rate; *ASDR* age-standardized death rate; *DALYs* disability-adjusted life years; *ASR* age-standardized rate

**Fig. 1:**
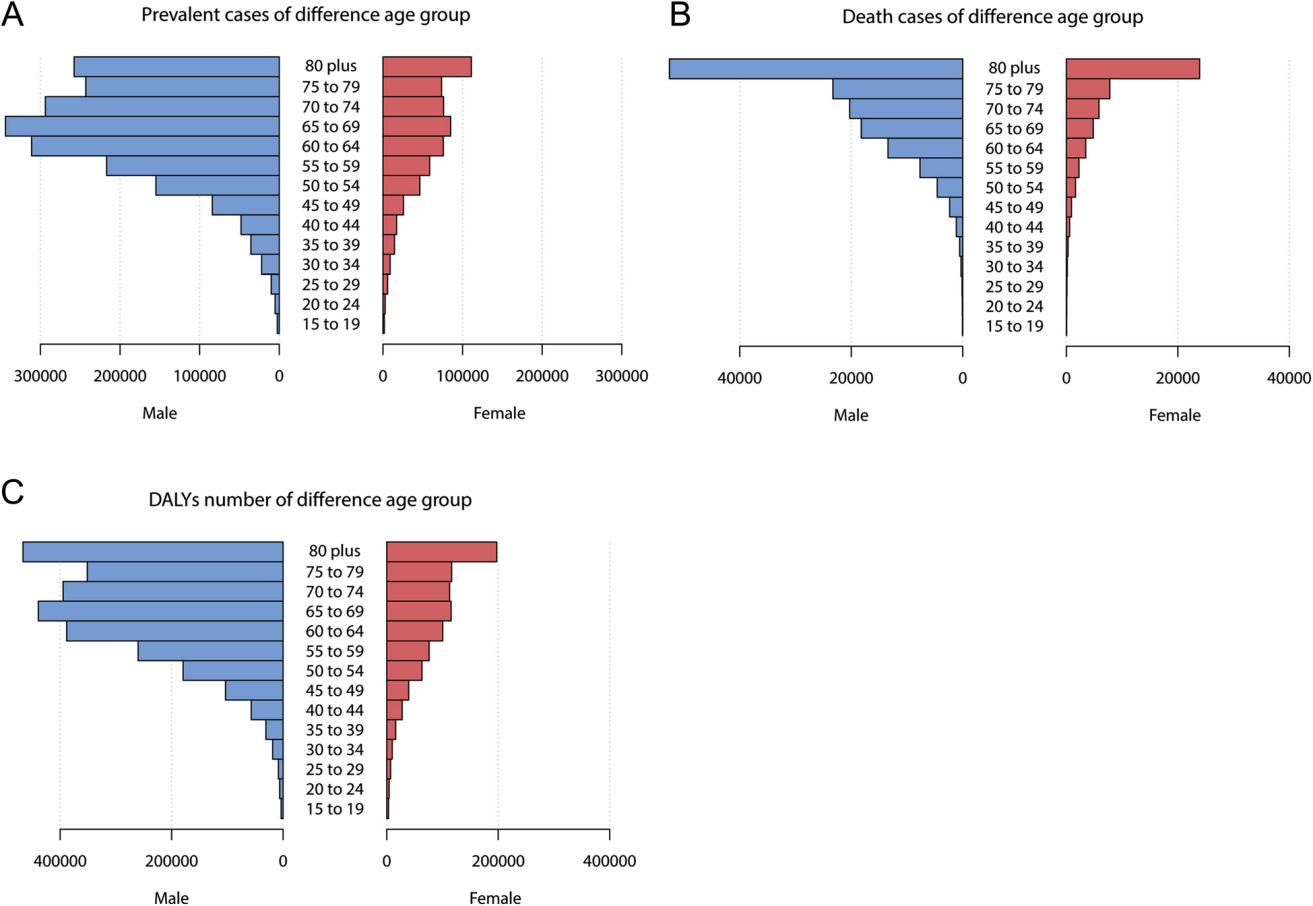
Contribution of different age group for male and female to absolute bladder cancer prevalent cases (**a**), death number (**b**), and DALYs number (**c**) globally in 2017. DALYs,disability-adjusted life years

**Fig. 2:**
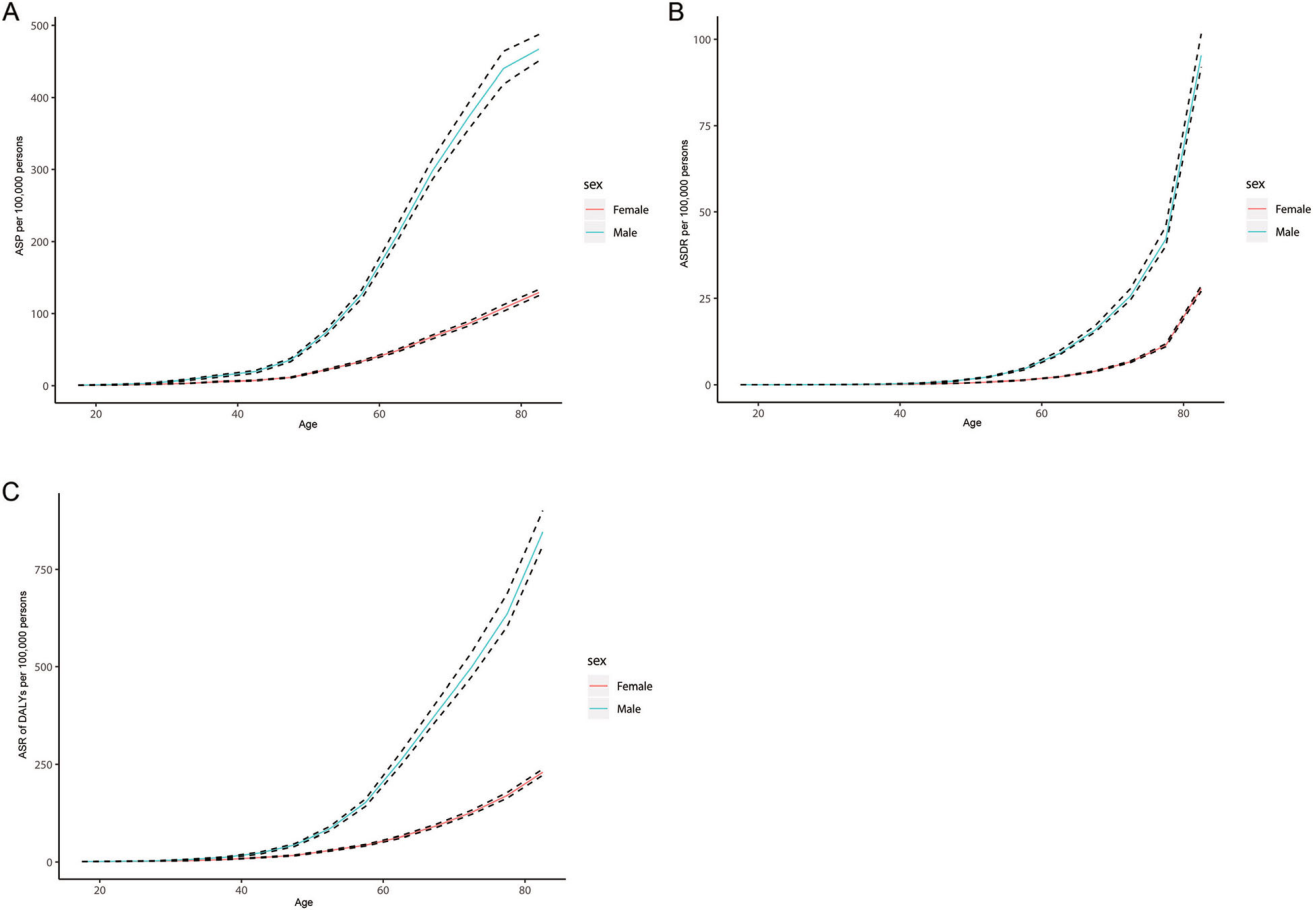
Age-standardised prevalence (**a**), mortality (**b**) and DALYs (**c**) rates of bladder cancer in 2017, by age and sex. DALYs, disability-adjusted life years

Figure 1 shows the prevalent cases, mortality, and DALYs of BCa according to sex and age group. Within any age group, the value of each of the three indicators was higher in males than in females, with similar trends in males and females. Among both sexes the prevalence was highest in people older than 50 years, increased from 15 to 69 years, decreased slightly from 70 to 79 years, and then increased further in those aged > 79 years. According to sex, the prevalence was highest in females aged > 79 years and males aged 65–69 years (Fig. 1a). The number of deaths increased with age, and was far larger in those aged > 79 years than in the other age groups (Fig. 1b). The trend for DALYs of BCa was similar to that for prevalence, with the difference being that the number of DALYs was largest among those aged > 79 years in both males and females (Fig. 1c). Figure 2 shows the trends of ASP, ASDR, and ASR of DALYs of BCa for the two sexes in the different age groups. All three indicators increased with age in both males and females (Fig. 2).

### Burden of BCa at regional and national levels

Among the 21 regions included in GBD 2017, the number of BCa cases was largest in Western Europe (684,364, 95% CI = 650,758–718,768) and East Asia (450,488, 95% CI = 426,143–507,850), as were the numbers of deaths due to BCa (43,838, 95% CI = 42,016–45,622; and 32,750, 95% CI = 31,030–37,532; respectively) and DALYs of BCa (659,027, 95% CI = 625,700–693,024; and 621,440, 95% CI = 585,238–715,420; respectively). Meanwhile, Oceania had the smallest numbers of cases (1129, 95% CI = 1321–944), deaths (102, 95% CI = 83–116), and DALYs (2825, 95% CI = 2333–3309) (Table 1).

The ASP of BCa was highest in Western Europe (80.17/100,000 persons, 95% CI = 76.12–84.12/100,000 persons) and Central Europe (61.55/100,000 persons (95% CI = 59.46–63.31/100,000 persons), as were the ASDR (4.21/100,000 persons, 95% CI = 4.02–4.38/100,000 persons; and 4.50/100,000 persons, 95% CI = 4.34–4.67/100,000 persons, respectively) and the ASR of DALYs (73.07/100,000 persons, 95% CI = 69.24–77.13/100,000 persons; and 87.47/100,000 persons, 95% CI = 83.82–91.32/100,000 persons; respectively). The ASP of BCa was lowest for South Asia (10.57/100,000 persons, 95% CI = 9.86–11.88/100,000 persons) and Andean Latin America (12.38/100,000 persons, 95% CI = 11.00–13.95/100,000 persons). The ASDR was lowest for Central Latin America (1.36/100,000 persons, 95% CI = 1.30–1.42/100,000 persons) and for Andean Latin America (1.40/100,000 persons, 95% CI = 1.26–1.60/100,000 persons), as was the ASR of DALYs (25.04/100,000 persons, 95% CI = 23.96–26.21/100,000 persons; and 24.58/100,000 persons, 95% CI = 21.90–27.87/100,000 persons; respectively) (Table 1).

**Fig. 3:**
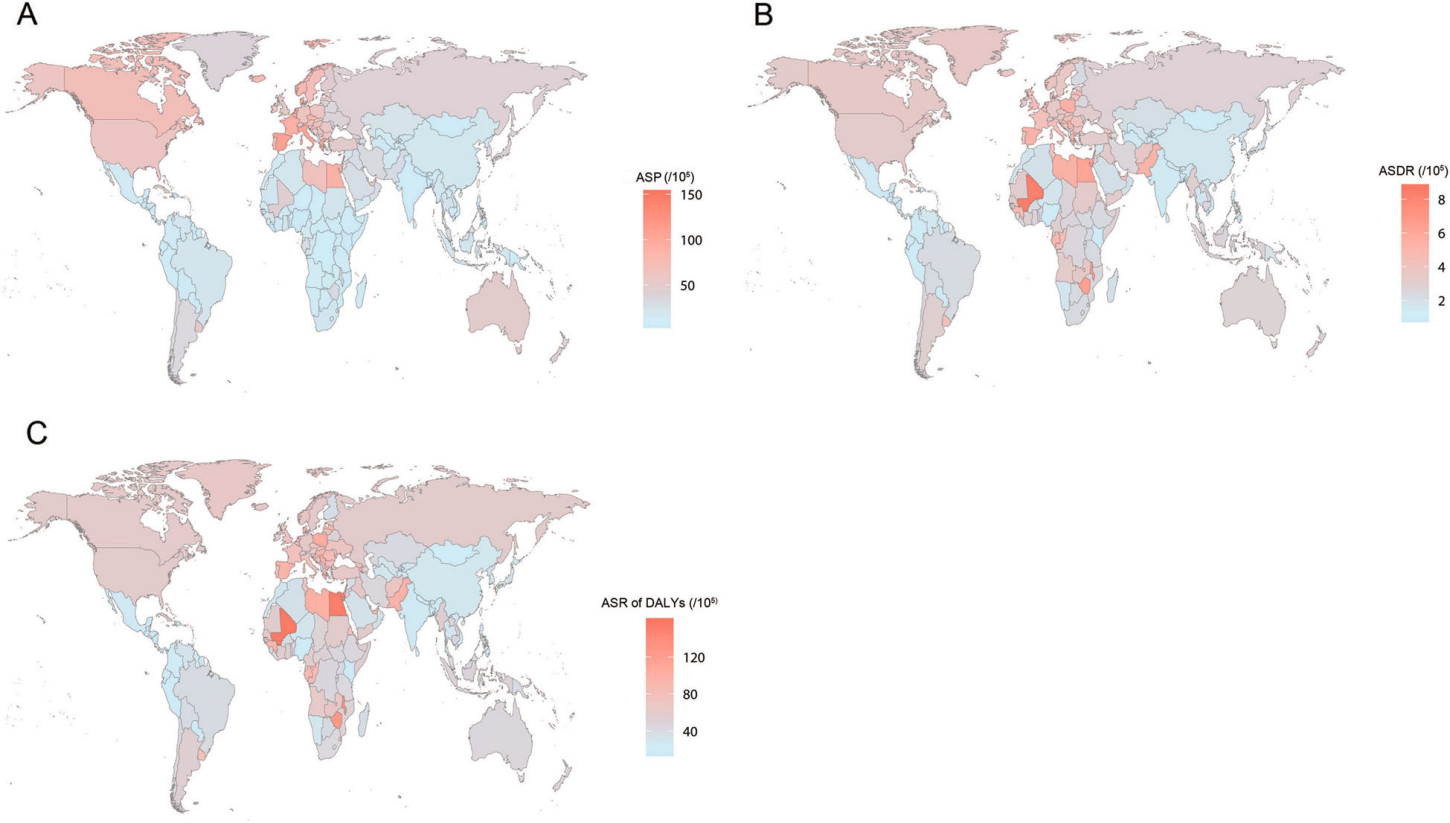
Age-standardised prevalence (**a**), mortality (**b**) and DALYs (**c**) rates of bladder cancer in 195 countries or territories in 2017. DALYs, disability-adjusted life years. The figure is our own and is drawn using R software (version 3.5.1; https://www.r-project.org/) by loading the “maps” and “ggplots2” packages

Among the 50 most-populous countries globally, the ASP of BCa was highest in Spain (107.48/100,000 persons, 95% CI = 97.75–118.63/100,000 persons), followed by Italy, and lowest in Kenya (6.76/100,000 persons, 95% CI = 5.49–7.91/100,000 persons), followed by Bangladesh. Egypt showed the highest ASDR of BCa (6.18/100,000 persons, 95% CI = 4.71–9.28/100,000 persons) and ASR of DALYs (153.66/100,000 persons, 95% CI = 121.00–205.32/100,000 persons), followed by Poland. Kenya had the lowest ASDR of BCa (1.08/100,000 persons, 95% CI = 0.86–1.28/100,000 persons), followed by Bangladesh. Bangladesh showed the lowest ASR of DALYs (19.33/100,000 persons, 95% CI = 14.48–31.65/100,000 persons) (Supplementary Table 1, Fig. 3).

### Relationship between BCa burden and SDI

Among the five GBD SDI quintiles, high-SDI countries showed the highest ASP of BCa (62.51/100,000 persons, 95% CI = 60.51–64.54/100,000 persons), ASDR (3.44/100,000 persons, 95% CI = 3.34–3.53/100,000 persons), and ASR of DALYs (59.66.81/100,000 persons, 95% CI = 57.40–62.14/100,000 persons), followed by countries with high-middle and low-middle SDIs. Low-SDI countries had the lowest ASP (10.16/100,000 persons, 95% CI = 9.28–12.00/100,000 persons), while middle-SDI countries had the lowest ASDR (1.69/100,000 persons, 95% CI = 1.61–1.88/100,000 persons) and ASR of DALYs (30.81/100,000 persons, 95% CI = 29.05–33.88/100,000 persons) (Table 1).

**Fig. 4:**
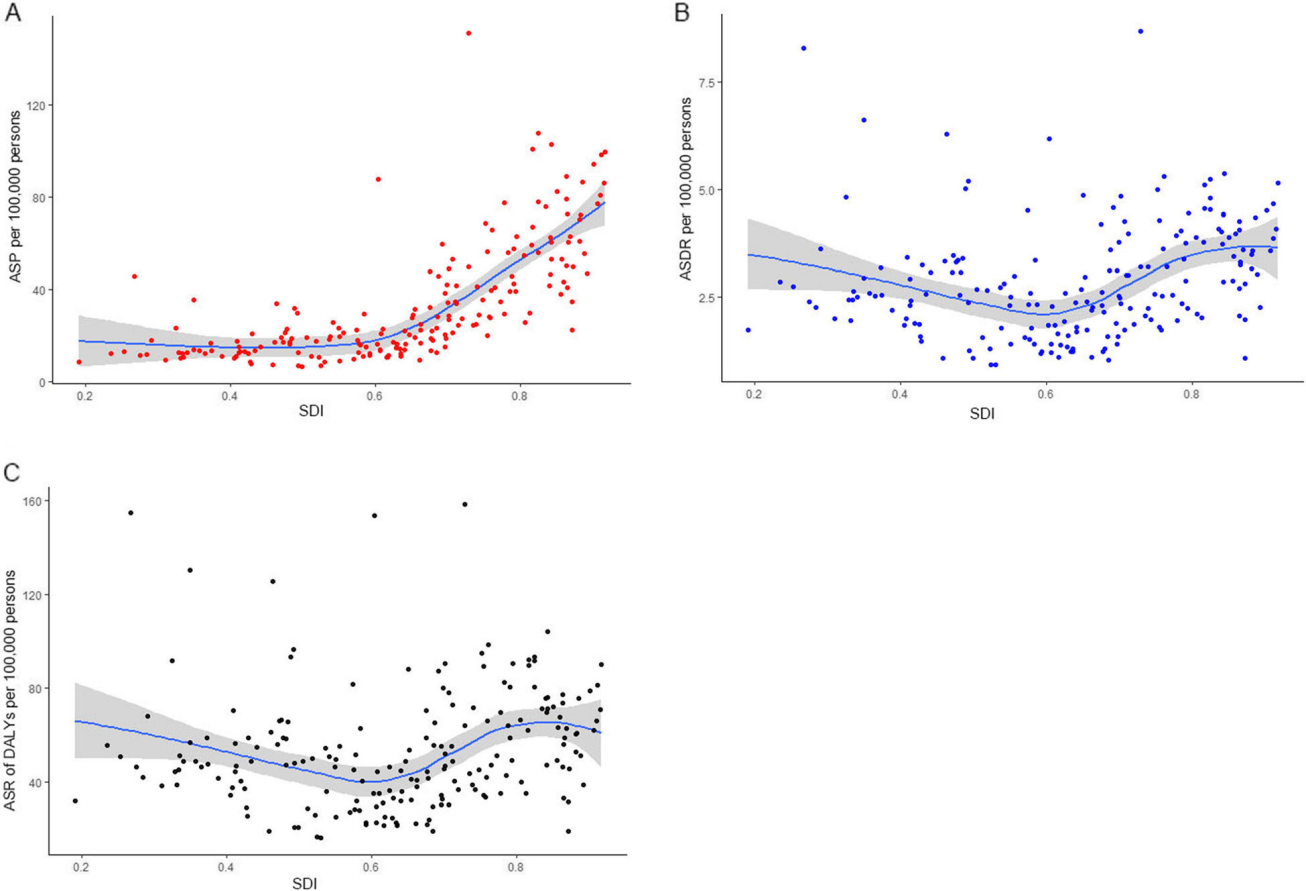
The correlation between age-standardised prevalence (**a**), mortality (**b**) and DALYs (**c**) rates of bladder cancer and SDI of 195 countries or territories in 2017. The fitted curve and 95% CI are obtained by locally weighted regression. DALYs,disability-adjusted life years

We also analyzed the relationships of ASP, ASDR, and ASR of DALYs of BCa with SDI among the 195 included countries or territories, which revealed positive correlations for all three parameters (*P* < 0.0001, *ρ* = 0.68; *P* = 0.0048, *ρ* = 0.20; and *P* = 0.043, *ρ* = 0.15 for ASR of DALYs; respectively)(Fig. 4).

### Attributable risk factors of BCa burden

The attributable risk factors for the BCa burden were smoking and high fasting plasma glucose. Globally, 33.72% (95% CI = 25.66–41.10%) deaths and 36.80% (95% CI = 28.49–43.66%) DALYs caused by BCa could be attributed to smoking. These varied by SDI quintiles, with high-middle-SDI countries showing the highest percentage of smoking-attributable BCa deaths (39.47, 95% CI = 30.58–47.05%) and DALYs of BCa (42.96, 95% CI = 34.26–50.23%), and low-SDI countries showing the lowest percentages (23.22, 95% CI = 14.82–29.95%; and 22.75, 95% CI = 14.01–29.42%, respectively). Globally, 9.87% (95% CI = 2.06–21.20%) deaths and 8.91% (95% CI = 1.81–19.27%) DALYs caused by BCa could be attributable to high fasting plasma glucose. There were few variations in the percentages of deaths and DALYs caused by high fasting plasma glucose among the five SDI quintiles.

## Discussion

We have presented the latest data and patterns of global prevalence, mortality, and DALYs of BCa based on GBD 2017. Overall, the prevalence and mortality of BCa were higher in males (especially older males), and also showed geographical variations, being higher in Western Europe and Central Europe, and lower in Andean Latin America, Central Latin America, and South Asia. In addition, the prevalence and mortality of BCa were positively associated with the SDI, with high-SDI countries showing the highest prevalence and mortality rates of BCa. Smoking was the main risk factor for the BCa burden.

The prevalence and DALYs rate of all age groups over 70 years old was higher than that of 65–69 years old group. However, the number of patients and DALYs of those groups were less than those of 65–69 years old group. These indicated that the number of people in each age group over 70 years old was smaller than that of 65–69 years old group, which is consistent with the World Bank’s survey results. According to the World Bank’s data, the mean life expectancy of the world population in 2016 is 72.04 years old (https://ourworldindata.org/grapher/life-expectancy-at-birth-total-years). That is, the number of people aged 70–74 years old may be less than the number of people aged 65–69 years old.

The prevalence of BCa appears to vary across world regions, which might be correlated with the distribution of its risk factors: smoking and occupational exposures to carcinogens in developed countries, and chronic bladder urothelial irritation from Schistosoma haematobium infection in Africa and the Middle East [11]. In general, the estimates for BCA from the GBD 2017 data are consistent with the data in the Global Cancer Observatory (http://gco.iarc.fr), which indicates that the prevalence of BCa is higher in Western Europe, Central Europe, North America, Turkey, Egypt, and Mali, while the mortality rate of BCa is higher in Western Europe, Central Europe, North Africa, Mali, and Turkey. The differences in the prevalence and mortality rates of BCa between North America and North Africa could be due to differences in medical conditions. One-third of BCa patients had muscle-invasive disease, which is more serious and has a higher mortality rate than non-muscle-invasive BCa [12]. There were no major improvements in muscle-invasive BCa management during recent decades until recent years; immunotherapy has been shown to increase survival, with responses in 20–30% of the patients presenting with advanced and metastatic BCa [13]. However, immunotherapy is an expensive treatment and has certain requirements for medical conditions, which may result in the benefits in controlling the mortality of BCa being greater in developed countries.

The burden of BCa also differs markedly between regions with different economic and social development statuses. Especially for prevalence, a previous study found that more than half of all BCa cases occur among the 20% of the world population living in countries with a very high Human Development Index (HDI; based on health, education, and income), while only 5% of all diagnoses occur in those with a low HDI. In terms of mortality, there is somewhat less variation between parts of the world with different HDIs [14]. The present study reached similar conclusions for BCa results obtained from GBD 2017 data: after dividing the world into five parts according to SDI quintiles, almost half of the cases were distributed in high-SDI countries, while the BCa patients in low-SDI countries accounted for fewer than 3% of all cases. The ASP showed the same pattern, being 62/100,000 and 10/100,000 in high- and low-SDI countries, respectively. This phenomenon might be attributable to higher incidence and longer survival time in high-SDI countries. The incidence of BCa is higher in older males, and population aging in developed countries is more pronounced in developed countries than in less-developed countries, [15] which has led to more BCa patients being diagnosed in developed countries. In addition, the survival period of BCa is longer in developed countries due to better medical conditions [6]. These two reasons cause higher prevalence in high-SDI areas. However, despite this, the ASDR of BCa did not show such a marked difference between high- and low-SDI countries, at 3.4/100,000 and 1.9/100,000, respectively. The GBD Collaborators pointed out that high- and high-middle-SDI countries generally have higher Healthcare Access and Quality Indexes and benefit more for noncommunicable diseases [16]. This may explain why despite the prevalence of BCa being much higher in high-SDI countries than in low-SDI countries, the associated mortality rate is only slightly higher. Our correlation analysis also found that the ASP, death rate, and DALYs of BCa were positively correlated with the SDI value of each country, with the correlation being stronger for the prevalence rate (correlation coefficient of 0.68).

Tobacco smoking is the main identified risk factor for BCa, accounting for 50–65% of the urothelial cell carcinoma cases in males and for 20–35% of those in females [17]. A large cohort study found that the risks of BCa in current and former smokers were 4.06- and 2.22-fold higher, respectively, than in those who had never smoked [18]. In the present study we also found the main attributable risk factor for the BCa burden was smoking. While the effect of smoking on the BCa burden is more obvious in high-SDI countries than in low-SDI countries, this may be because low-SDI countries are mostly concentrated in Africa, and the histological subtype of BCa in most parts of Africa has historically been squamous cell carcinoma linked to schistosomiasis [19]. Stopping smoking is the most-effective way to reduce the BCa burden, possibly protecting 30% of males and 28% of the population aged < 65 years from the incidence of BCa [20].

Several articles also describe the global burden of BCa. Richters et al. provided an overview of the burden of BCa worldwide using published data and some public database [6]. The main purpose of the document is to describe and summarize the general laws of BCa epidemiology on a global scale, and for different indicators and regions, data from different years are used. They provided some major summary which was similar to the conclusion of our research. The main difference in our article is that that the data used is from the same source and is 2017 data. We mapped the global burden of BCa in 2017. Dy et al. used GBD 2013 data to describe the incidence and mortality of BCa, and risk factor-attributable BCa deaths from 1990 to 2013 [9]. Ebrahimi et al. described the above indicators and DALY of BCa from 1990 to 2016 using GBD 2016 data [8]. In this study, we describe the BCa burden in 2017, which can be considered as a supplement to these two articles. After all, the GBD database is constantly being updated. Our study found compared with data in 2016, the ASDR and DALY rate of BCa in 2017 have decreased, but the absolute number is still increasing. The other difference lies in the different research indicators. This study described the prevalence of BCa, which is not described in the published two articles. The results of this study should be explained combined with the two existing articles of GBD data, so that we can more clearly understand the changes in the burden of BCa in the past 28 years (1990–2017).

Based on GBD 2017 data, we have described the burden of BCa across countries and regions globally, which may facilitate the understanding of the disease status. However, this study had some limitations. Firstly, muscle-invasive BCa is more serious and is the main type responsible for BCa-related deaths. The data of its burden are more clinical meaningful, but GBD 2017 did not provide detailed information. Secondly, as several African countries undergo social and economic development and changes, the histological subtype of BCa may gradually shift from squamous cell carcinoma to transitional cell carcinoma [21, 22]. Moreover, the burdens attributable to different pathological subtypes of BCa were not estimated separately, which meant that we cannot know whether the pathological type of BCa has changed in Africa countries. Thirdly, despite the application of methods for reducing bias in GBD-2017-based estimates, including correcting for misclassifications and incompleteness, and redistributing garbage codes, the low quality of data sources in several countries may have led to data inaccuracy.

## Conclusion

In summary, in 2017 BCa remained a cancer that is diagnosed predominantly in males. The burden associated with BCa mainly occurs in people older than 60 years, varies across the world, and is significantly associated with development level. The prevalence and mortality rates of BCa are higher in developed regions. Smoking-cessation strategies are the most-effective way to control this burden, and policymakers should therefore strengthen these strategies.

## Supplementary Information

**Additional file 1.** The prevalent cases, death number and DALYs and their age-standardized rate of bladder cancer in 2017.

## Data Availability

The raw data of this study can be found though the Global Health Data exchange software (http://www.healthdata.org/gbd/).

## Abbreviations

BCa: Bladder cancer
GBD: Global Burden of Disease study
DALYs: Disability-adjusted life years
ASR: Age-standardized rate
ASP: Age-standardized prevalence
ASDR: Age-standardized death rate
SDI: Sociodemographic index
HDI: Human Development Index
