# Supplementary material for: Global, regional, and national burdens of bladder cancer in 2017: estimates from the 2017 global burden of disease study
Source: BMC Public Health. 2020 Nov 11;20:1693. doi: 10.1186/s12889-020-09835-7 (PMC7659112; doi:10.1186/s12889-020-09835-7)
Supplement: Supplementary file 1 — Additional file 1. The prevalent cases, death number and DALYs and their age-standardized rate of bladder cancer in 2017. [file 12889_2020_9835_MOESM1_ESM.pdf]

Global, regional, and national burdens of bladder cancer in 2017: estimates from the 2017 Global Burden of Disease study

Hairong He(M.M.)[1,2], Hongjun Xie(M.M.)[3], Yule Chen(M.M.)[3], Chengzhuo Li (M.S.)[2], Didi Han (M.S.)[2], Fengshuo Xu (M.S.)[2], Jun Lyu(M.D.;Ph.D.)[1,2,4,*]

Table of contents

Supplementary Table 1. The prevalent cases, death number and DALYs and their age-standardized rate of bladder cancer in 2017

Supplementary Table 1. The prevalent cases, death number and DALYs and their age-standardized rate of bladder cancer in 2017.

**Region**	**Location**	**Prevalent cases (No.×100)**	**ASP (per 100,000 persons)**	**Death number (No.×100)**	**ASDR (per 100,000 persons)**	**DALYs number (No.×100)**	**ASR of DALYs (per 100,000 persons)**
Andean Latin America	Bolivia	13.01(10.45-16.24)	15.06(12.12-18.65)	1.78(1.45-2.2)	2.32(1.89-2.86)	34.18(27.36-42.73)	40.41(32.52-50.34)
	Ecuador	16.7(14.94-18.61)	11.21(10.05-12.49)	1.81(1.63-2.01)	1.29(1.17-1.44)	31.3(28.2-34.7)	21.38(19.26-23.65)
	Peru	37.58(31.69-45.54)	12.22(10.3-14.87)	3.78(3.21-4.69)	1.23(1.04-1.53)	66.37(55.77-81.89)	21.78(18.26-26.96)
Australasia	Australia	199.57(176.2-224.4)	49.91(44.11-56.18)	12.71(11.4-14.16)	2.8(2.51-3.12)	188.61(167.84-211.31)	45.64(40.49-51.2)
	New Zealand	34.36(31.18-37.7)	46.34(42.18-50.84)	2.34(2.14-2.55)	2.85(2.6-3.1)	36.19(32.84-39.62)	47.3(43.01-51.64)
Caribbean	Antigua and Barbuda	0.25(0.23-0.27)	24.7(22.63-26.89)	0.02(0.02-0.02)	2.25(2.05-2.44)	0.4(0.37-0.43)	40.25(36.83-43.63)
	Barbados	1.18(1.06-1.32)	24.83(22.25-27.63)	0.11(0.1-0.12)	2.24(2.03-2.48)	1.89(1.7-2.1)	38.98(34.99-43.21)
	Belize	0.44(0.4-0.48)	16.3(14.96-17.8)	0.04(0.04-0.05)	1.84(1.67-2.01)	0.91(0.84-1)	35.12(32.12-38.47)
	Bermuda	0.67(0.61-0.74)	54.82(49.87-60.23)	0.05(0.05-0.06)	3.89(3.56-4.29)	0.84(0.76-0.92)	66.55(60.54-73.33)
	Cuba	83.56(74.39-94.99)	45.51(40.45-51.69)	7.01(6.26-7.89)	3.61(3.22-4.06)	121.94(108.61-138.32)	65.3(58.14-74.24)
	Dominica	0.26(0.23-0.28)	28.31(25.79-31.14)	0.03(0.03-0.03)	3.12(2.85-3.41)	0.52(0.47-0.57)	55.72(50.63-61.22)
	Dominican Republic	10.52(8.39-13.94)	11.32(9.04-14.95)	1.06(0.86-1.37)	1.2(0.98-1.55)	19.98(15.97-26.63)	21.91(17.52-29.17)
	Grenada	0.32(0.3-0.35)	21.91(20.11-23.82)	0.04(0.04-0.04)	2.38(2.19-2.59)	0.68(0.63-0.74)	45(41.52-49.14)
	Guyana	0.7(0.61-0.79)	11.05(9.74-12.45)	0.08(0.07-0.09)	1.4(1.25-1.57)	1.71(1.51-1.93)	27.8(24.63-31.22)
	Haiti	9.72(7.58-12.93)	15.07(11.68-19.86)	1.62(1.25-2.12)	3.08(2.35-3.98)	34.96(27.06-46.59)	54.74(42.19-72.04)
	Jamaica	6.4(5.21-7.98)	22.18(18.03-27.7)	0.63(0.52-0.77)	2.15(1.76-2.65)	11.99(9.76-14.99)	41.8(34.01-52.39)
	Puerto Rico	19.71(17.93-21.64)	29.84(27.11-32.71)	1.58(1.44-1.72)	2.02(1.84-2.21)	24.58(22.25-27.02)	35.36(32.09-38.88)
	Saint Lucia	0.61(0.56-0.66)	29.04(26.67-31.61)	0.06(0.06-0.07)	2.95(2.71-3.21)	1.12(1.03-1.22)	53.84(49.36-58.74)

	Saint Vincent and the Grenadines	0.28(0.26-0.31)	20.67(18.84-22.68)	0.03(0.03-0.03)	2.29(2.09-2.53)	0.61(0.55-0.67)	44.54(40.56-49)
	Suriname	0.82(0.72-0.93)	13.96(12.35-15.81)	0.09(0.08-0.1)	1.7(1.51-1.9)	1.85(1.64-2.09)	31.98(28.32-36.24)
	The Bahamas	0.77(0.69-0.85)	19.9(17.91-21.95)	0.06(0.05-0.07)	1.81(1.62-1.99)	1.28(1.15-1.42)	34.38(30.81-37.96)
	Trinidad and Tobago	3.38(2.75-4.11)	18.74(15.23-22.87)	0.28(0.23-0.33)	1.59(1.32-1.91)	5.91(4.82-7.22)	32.69(26.66-39.94)
	Virgin Islands, U.S.	0.49(0.4-0.57)	25.9(21.24-30.14)	0.04(0.03-0.05)	2.1(1.71-2.41)	0.77(0.62-0.9)	40.02(32.44-46.22)
Central Asia	Armenia	20.54(18.99-22.3)	48.95(45.38-52.95)	2.02(1.87-2.17)	4.85(4.49-5.21)	38.07(35.31-41.03)	90.7(84.11-97.91)
	Azerbaijan	27.83(23.9-32.08)	26.86(23.59-30.5)	2.04(1.78-2.4)	2.48(2.15-3.05)	50.15(43.49-57.46)	52.13(45.84-59.76)
	Georgia	23.91(21.89-26.14)	41.86(38.43-45.76)	2.31(2.12-2.51)	3.78(3.48-4.11)	47.04(43.14-51.43)	80.01(73.32-87.56)
	Kazakhstan	44.06(39.55-49.07)	24.5(22.12-27.11)	3.28(2.97-3.6)	2.07(1.89-2.27)	75.65(67.77-84.33)	43.75(39.38-48.48)
	Kyrgyzstan	6.51(5.6-7.42)	13.73(11.93-15.56)	0.56(0.49-0.63)	1.4(1.23-1.57)	13.17(11.34-15.02)	29.55(25.67-33.38)
	Mongolia	2.28(1.98-2.62)	9.3(8.07-10.6)	0.2(0.17-0.23)	1.11(0.91-1.29)	5.01(4.29-5.74)	22.21(18.71-25.6)
	Tajikistan	6.34(5.43-8.51)	10.75(9.19-15.32)	0.58(0.48-0.87)	1.3(1.09-1.98)	14.04(11.82-20.47)	25.98(21.6-39.72)
	Turkmenistan	6.58(5.87-7.35)	15.32(13.75-16.99)	0.48(0.43-0.53)	1.4(1.26-1.54)	12.15(10.77-13.51)	30.25(26.9-33.46)
	Uzbekistan	41.54(36.24-47.37)	16.43(14.54-18.61)	2.99(2.64-3.38)	1.61(1.43-1.8)	76.23(66.45-87.19)	33.18(29.28-37.58)
Central Europe	Albania	5.17(4.18-6.48)	12.61(10.2-15.63)	0.43(0.35-0.58)	1.03(0.83-1.36)	8.03(6.32-10.53)	19.06(15.09-24.78)
	Bosnia and Herzegovina	25.11(20.17-28.2)	41.46(33.33-46.41)	2.41(1.98-2.7)	3.98(3.26-4.43)	44.8(36.18-50.63)	72.82(58.71-81.88)
	Bulgaria	78.98(72.31-86.65)	57.71(52.79-63.05)	5.83(5.35-6.36)	3.75(3.45-4.08)	115.47(105.22-126.33)	80.37(73.36-87.65)
	Croatia	65.76(60.78-71.38)	77.77(71.83-84.46)	4.34(4.02-4.68)	4.55(4.23-4.89)	72.1(66.6-78.2)	80.37(74.2-87.18)
	Czech Republic	168.78(154.91-184.03)	82.23(75.42-89.71)	8.53(7.89-9.2)	3.89(3.6-4.18)	151.94(139.42-165.13)	71.99(65.95-78.25)
	Hungary	122.87(114.02-131.81)	67.05(62.3-71.88)	9.29(8.69-9.97)	4.56(4.27-4.89)	172(160.2-184.9)	89.99(83.74-96.85)
	Macedonia	18.99(16.28-21.75)	56.2(47.96-64.45)	1.47(1.26-1.71)	4.27(3.66-5.01)	30.61(26.21-35.14)	89.42(76.4-102.37)

	Montenegro	4.58(3.99-5.22)	45.87(40.07-52.05)	0.34(0.3-0.39)	3.39(2.96-3.88)	6.51(5.68-7.42)	64.1(56.16-73.02)
	Poland	412.15(380.12-445.92)	60.53(55.75-65.23)	39.12(36.38-41.89)	5.37(4.98-5.76)	725.35(669.44-784.16)	104.13(95.76-112.6)
	Romania	194.59(179.23-210.53)	55.86(51.44-60.49)	15.8(14.66-16.92)	4.04(3.76-4.32)	302.24(279.38-326.28)	82.68(76.4-89.21)
	Serbia	107.27(78.96-122.22)	68.63(49.72-77.81)	8.35(6.28-9.41)	5.01(3.77-5.64)	153.92(112.33-175.39)	94.97(68.74-108.02)
	Slovakia	57.12(49.98-63.74)	62.28(55.11-69.29)	3.42(2.76-3.82)	3.73(3.02-4.17)	64.35(54.58-72.59)	69.78(59.56-78.28)
	Slovenia	17.47(15.96-19.14)	43.37(39.57-47.66)	1.87(1.7-2.05)	3.98(3.63-4.36)	29.14(26.39-32.03)	67.65(61.34-74.39)
Central Latin America	Colombia	76.24(67.14-85.8)	14.16(12.45-15.95)	6.74(6-7.52)	1.24(1.11-1.39)	122.45(107.91-138.24)	22.77(20.08-25.7)
	Costa Rica	12.31(11.13-13.68)	25.05(22.71-27.79)	1.05(0.95-1.18)	2.21(1.99-2.48)	18.36(16.64-20.31)	37.98(34.48-42.09)
	El Salvador	7.03(5.79-8.5)	12.33(10.15-14.93)	0.72(0.61-0.85)	1.24(1.05-1.48)	12.96(10.7-15.58)	22.75(18.78-27.39)
	Guatemala	8.09(7.22-8.97)	7.06(6.3-7.83)	0.92(0.83-1.03)	0.93(0.83-1.03)	18.48(16.48-20.58)	16.7(14.94-18.58)
	Honduras	6.97(5.59-8.52)	11.18(9.03-13.61)	0.86(0.71-1.04)	1.56(1.29-1.89)	17.34(13.94-21.09)	28.84(23.35-34.96)
	Mexico	171.83(164.51-181.03)	14.77(14.15-15.55)	14.39(13.79-15.11)	1.34(1.29-1.41)	283.2(270.5-299.01)	24.89(23.78-26.27)
	Nicaragua	3.96(3.47-4.64)	8.5(7.45-9.95)	0.4(0.35-0.45)	0.92(0.81-1.04)	7.3(6.38-8.5)	16.23(14.19-18.81)
	Panama	5.35(4.91-5.85)	13.52(12.43-14.81)	0.49(0.44-0.53)	1.22(1.11-1.33)	8.6(7.86-9.4)	21.83(19.97-23.85)
	Venezuela	55.34(47.53-64.34)	19.3(16.61-22.41)	4.44(3.85-5.12)	1.74(1.51-1.99)	89.94(77.46-104.95)	32.46(27.94-37.94)
Central Sub-Saharan Africa	Angola	17.71(12.83-28.85)	17.12(12.18-28.7)	2.57(1.82-4.2)	3.34(2.31-5.65)	61.39(44.1-98.89)	61.12(43.48-100.21)
	Central African Republic	2.18(1.46-3.05)	10.79(7.79-14.07)	0.41(0.29-0.54)	2.63(2.02-3.24)	10.42(6.99-14.62)	51.13(36.82-67.26)
	Congo	5.44(3.98-7.88)	22.58(16.23-33.64)	0.83(0.59-1.25)	4.52(3.09-7.13)	19.1(13.95-27.9)	81.91(58.45-123.17)
	Democratic Republic of the Congo	39.61(30.67-50.36)	12.14(9.5-15.85)	6.45(5-8.4)	2.53(1.94-3.39)	149.15(115.42-189.71)	46.37(36-59.87)
	Equatorial Guinea	0.8(0.53-1.16)	17.1(11.84-23.97)	0.09(0.07-0.12)	2.59(1.9-3.41)	2.03(1.38-2.9)	46.5(32.63-63.84)
	Gabon	2.88(1.89-5.29)	27.94(18.14-52.34)	0.42(0.26-0.8)	4.86(2.99-9.54)	8.87(5.82-16.33)	88.33(57.32-164.92)
East Asia	China	4181.08(3943.12-4736.91)	21.41(20.2-24.11)	306.43(289.21-353.08)	1.75(1.65-2.01)	5824.84(5475.46-6724.92)	30.53(28.72-35.08)

	North Korea	61.85(51.81-72.78)	19.47(16.36-22.77)	5.28(4.37-6.2)	1.79(1.48-2.11)	112.66(94.84-131.45)	35.8(30.25-41.78)
	Taiwan (Province of China)	189.38(175.77-204.7)	50.1(46.61-54.1)	10.51(9.83-11.2)	2.71(2.53-2.89)	176.79(164.48-190.08)	46.36(43.08-49.83)
Eastern Europe	Belarus	54.34(46.38-62.59)	34.61(29.53-39.88)	4.18(3.66-4.71)	2.54(2.21-2.87)	82.09(69.89-94.65)	51.14(43.51-58.9)
	Estonia	13.35(11.37-15.55)	52.96(44.76-62.14)	1.02(0.89-1.17)	3.44(2.97-3.95)	16.91(14.39-19.73)	63.38(53.64-75.11)
	Latvia	21.01(18.41-23.9)	55.69(48.33-63.55)	2.12(1.87-2.39)	4.8(4.24-5.42)	36.68(32.08-41.99)	91.65(79.39-105.65)
	Lithuania	22.64(20.81-24.42)	41.54(38.09-44.99)	2.58(2.38-2.78)	4.01(3.69-4.31)	43.75(39.99-47.6)	75.56(68.77-82.44)
	Moldova	18.17(16.69-19.65)	32.15(29.54-34.8)	1.51(1.39-1.63)	2.62(2.42-2.83)	33.77(31-36.68)	58.93(54.14-63.99)
	Russian Federation	972.7(935.45-1008.33)	42.48(40.91-44.04)	68.24(66.71-69.74)	2.87(2.81-2.93)	1376.6(1333.62-1422.89)	58.84(56.99-60.84)
	Ukraine	305.02(280.17-331)	41.09(37.68-44.65)	25.62(23.69-27.77)	3.2(2.97-3.46)	547.53(502.63-596.43)	71.6(65.75-78.1)
Eastern Sub-Saharan Africa	Burundi	3.81(2.53-5.05)	9.45(6.32-12.39)	0.63(0.43-0.83)	2.01(1.36-2.64)	15.32(10.15-20.32)	38.44(25.77-50.61)
	Comoros	0.62(0.45-0.79)	13.71(10.1-17.5)	0.1(0.07-0.13)	2.58(1.92-3.27)	2.17(1.59-2.78)	48.96(35.89-62.69)
	Djibouti	1.07(0.67-1.58)	18.9(12.23-27.03)	0.15(0.09-0.21)	3.4(2.3-4.73)	3.59(2.25-5.24)	65.76(42.46-93.84)
	Eritrea	4.1(3.1-5.67)	17.45(13.27-24.39)	0.6(0.46-0.84)	3.43(2.6-4.87)	16.05(12.16-22.06)	70.35(53.4-98.01)
	Ethiopia	48.57(33.47-60.4)	12.45(8.55-15.46)	7.98(5.38-9.97)	2.43(1.64-3.03)	174.21(117.32-218.53)	45.38(30.48-56.83)
	Kenya	14.67(11.95-17.19)	6.76(5.49-7.91)	1.83(1.47-2.18)	1.08(0.86-1.28)	42.36(34.12-50.16)	20.6(16.56-24.46)
	Madagascar	10.71(8.68-13.14)	10.14(8.27-12.26)	1.61(1.3-1.95)	1.96(1.6-2.38)	40.37(32.57-49.57)	38.98(31.66-47.46)
	Malawi	27.13(18.09-35.08)	35.6(23.69-45.59)	4.49(2.98-5.72)	6.63(4.35-8.43)	97.1(65.27-124.81)	130.3(87.71-167.18)
	Mozambique	13.94(10-19.69)	12.58(9.26-17.49)	2.2(1.62-3.03)	2.51(1.9-3.44)	52.74(37.68-74.7)	48.89(35.77-67.64)
	Rwanda	6.25(5.14-7.6)	10.94(8.95-13.25)	0.93(0.76-1.13)	1.99(1.63-2.46)	21.05(17.33-25.75)	37.75(30.92-45.82)
	Somalia	7.7(5.05-12.99)	12.34(8.18-20.51)	1.41(0.94-2.32)	2.85(1.92-4.68)	34.72(22.73-58.5)	55.54(36.96-91.32)
	South Sudan	4.23(2.91-6.09)	11.54(8.19-16.3)	0.7(0.5-0.99)	2.4(1.73-3.31)	16.8(11.52-24.23)	46.37(32.63-65.43)
	Tanzania	30.64(24.24-37.7)	12.72(10.18-15.55)	4.81(3.87-5.89)	2.31(1.89-2.82)	104.45(82.62-128.51)	44.35(35.28-54.42)
	Uganda	15.05(12.24-18.22)	11.22(9.14-13.44)	2.44(2-2.94)	2.21(1.81-2.63)	54.59(44.55-66.54)	41.6(33.96-50.24)
	Zambia	10.9(7.94-15.55)	17.18(12.46-24.55)	1.8(1.31-2.58)	3.47(2.52-4.99)	41.07(29.82-58.5)	66.13(47.88-93.99)

High-income Asia Pacific	Brunei	1.18(1.03-1.33)	38.02(32.94-43.27)	0.07(0.06-0.08)	2.88(2.4-3.29)	1.39(1.22-1.57)	49.09(42.24-55.8)
	Japan	1333.01(1262.57-1401.67)	40.64(38.19-43.03)	94.6(91.73-97.84)	2.06(1.99-2.14)	1208.77(1148.16-1270.07)	33.38(31.51-35.31)
	Singapore	15.37(13.56-17.27)	22.4(19.86-25.09)	0.69(0.63-0.76)	1.07(0.97-1.18)	12.88(11.43-14.4)	19.15(17.01-21.41)
	South Korea	297.39(263.78-332.16)	34.89(30.9-38.96)	16.11(14.54-17.9)	1.97(1.78-2.18)	268.85(239.49-302.13)	31.67(28.25-35.59)
High-income North America	Canada	459.02(421.9-501.97)	70.04(64.29-76.75)	25.51(23.58-27.63)	3.49(3.22-3.79)	407.58(375.48-444.93)	60.67(55.91-66.15)
	Greenland	0.27(0.2-0.32)	39.5(29.25-45.69)	0.02(0.02-0.02)	3.63(2.73-4.12)	0.44(0.32-0.51)	66.08(48.73-75.84)
	United States	3219.86(3120.27-3329.14)	60.26(58.33-62.32)	193.83(188.25-200.67)	3.32(3.23-3.44)	3205.19(3080.29-3336.47)	58.93(56.66-61.34)
North Africa and Middle East	Afghanistan	19.09(15.15-25.14)	18.14(14.93-22.8)	3.02(2.44-3.86)	3.62(2.84-4.45)	70.87(55.7-95.91)	68.19(55.5-87.47)
	Algeria	67.31(58.52-76.28)	20.35(17.6-23.17)	5.6(4.89-6.39)	1.9(1.65-2.18)	109.79(95.12-124.39)	34.22(29.73-38.89)
	Bahrain	3.06(2.61-3.78)	33.73(29.37-39.73)	0.17(0.15-0.19)	3(2.61-3.41)	3.79(3.23-4.6)	48.75(42.34-56.61)
	Egypt	648.81(490.45-904.78)	87.59(69.98-119.48)	34.65(26.99-46.3)	6.18(4.72-9.28)	1052.17(834.57-1432.44)	153.66(121-205.32)
	Iran	245.07(193.99-264.65)	34.24(27.45-36.84)	15.64(13.28-16.56)	2.53(2.17-2.67)	313.76(257.56-335.14)	45.77(38.03-48.93)
	Iraq	69.32(61.07-76.5)	29.56(26.07-32.38)	6.55(5.73-7.16)	3.36(2.91-3.68)	142.63(125.86-156.65)	62.92(55.51-68.87)
	Jordan	23.29(19.6-27.8)	38.2(32.36-45.54)	1.42(1.19-1.7)	3.11(2.59-3.71)	31.13(26.04-37.1)	55.11(46.21-65.79)
	Kuwait	9.91(8.27-11.63)	39.03(32.81-45.7)	0.46(0.39-0.53)	2.36(2.01-2.73)	9.99(8.39-11.68)	42.97(36.1-50.38)
	Lebanon	91.35(79.74-104.51)	150.98(130.75-173.11)	4.7(4.07-5.34)	8.69(7.52-9.84)	92.56(80.23-106.18)	158.63(136.93-182.38)
	Libya	30.94(24.98-38.5)	65.79(53.64-82.1)	2(1.64-2.43)	5.3(4.36-6.48)	43.12(35.27-53.23)	98.44(80.95-121.31)
	Morocco	51.31(40.29-63.65)	16.1(12.71-19.92)	5(3.95-6.08)	1.77(1.42-2.15)	98.66(77.18-121.3)	31.86(25.03-39.01)
	Oman	6.08(4.82-7.44)	29.13(23.63-35.06)	0.28(0.23-0.34)	1.97(1.63-2.33)	6.86(5.49-8.34)	36.91(29.96-44.23)
	Palestine	6.27(5.47-7.12)	25.59(22.29-29.05)	0.56(0.49-0.63)	2.81(2.42-3.18)	11.72(10.16-13.33)	50.7(43.88-57.52)
	Qatar	3.64(2.89-4.66)	40.92(33.27-50.17)	0.13(0.1-0.16)	3.2(2.41-3.87)	3.34(2.69-4.23)	47.23(37.91-57.78)
	Saudi Arabia	53.15(43.76-68.94)	29.51(25.17-37.13)	2.12(1.82-2.61)	1.97(1.7-2.35)	53.55(44.32-68.43)	35.05(30.15-43.25)
	Sudan	40.81(28.17-58.94)	22.74(15.38-33.05)	5.04(3.23-7.58)	3.36(2.09-5.1)	101.27(67.94-150.01)	58.75(38.62-87.29)
	Syria	30.64(24.77-39.26)	22.72(18.55-29.12)	2.03(1.66-2.63)	1.84(1.5-2.38)	45.23(36.65-57.9)	35.27(28.57-45.12)
	Tunisia	58.26(44.43-74.48)	47.71(36.55-60.82)	4.67(3.66-5.87)	4.19(3.3-5.22)	83.79(64.13-106.4)	70.5(54.28-89.18)

	Turkey	439.33(363.16-647.5)	49.8(41.19-73.7)	27.2(22.35-42.75)	3.2(2.62-5.05)	557.34(459.76-845.86)	64.1(52.77-97.75)
	United Arab Emirates	33.43(23.64-48.88)	62.73(43.26-103.56)	1.1(0.76-1.73)	4.45(3.09-7.41)	38.3(26.51-57.59)	90.5(61.95-151.89)
	Yemen	27.02(20.22-34.66)	20.94(15.5-26.84)	3.34(2.4-4.29)	3.25(2.32-4.13)	72.41(52.55-94.41)	58.9(42.45-75.62)
Oceania	American Samoa	0.14(0.11-0.16)	28.47(24.12-32.54)	0.01(0.01-0.01)	2.36(2.03-2.67)	0.21(0.17-0.24)	46.96(39.84-53.77)
	Federated States of Micronesia	0.14(0.1-0.17)	17.87(13.14-22.26)	0.01(0.01-0.02)	2.33(1.73-2.89)	0.32(0.24-0.4)	45.25(33.84-55.83)
	Fiji	1.33(1-1.57)	16.39(12.5-19.28)	0.11(0.08-0.13)	1.8(1.43-2.09)	2.7(2.04-3.2)	35.83(27.47-42.14)
	Guam	0.73(0.57-0.84)	39.12(30.97-44.98)	0.04(0.03-0.04)	2.25(1.93-2.52)	0.91(0.72-1.03)	49.3(39.83-56.04)
	Kiribati	0.06(0.05-0.07)	8.47(7.12-10.01)	0.01(0.01-0.01)	1.59(1.3-1.89)	0.19(0.16-0.23)	29.33(24.29-35.02)
	Marshall Islands	0.07(0.06-0.09)	19.65(16.02-24.12)	0.01(0-0.01)	2.5(2.12-2.91)	0.16(0.13-0.21)	49.52(40.85-59.74)
	Northern Mariana Islands	0.16(0.14-0.19)	28.14(24.38-31.89)	0.01(0.01-0.01)	1.74(1.55-1.97)	0.18(0.15-0.2)	33.4(29.42-37.64)
	Papua New Guinea	6.89(5.54-8.59)	13.09(10.89-15.68)	0.65(0.53-0.77)	1.89(1.51-2.23)	19(15.48-23.16)	40.59(33.42-48.34)
	Samoa	0.18(0.15-0.21)	12.61(10.65-14.94)	0.02(0.02-0.02)	1.53(1.27-1.79)	0.38(0.32-0.44)	28.39(23.74-33.32)
	Solomon Islands	0.44(0.35-0.54)	12.4(9.59-15.1)	0.05(0.04-0.06)	1.86(1.35-2.28)	1.22(0.94-1.5)	37.37(28.07-45.65)
	Tonga	0.14(0.1-0.18)	17.36(12.24-22.22)	0.01(0.01-0.02)	1.94(1.39-2.48)	0.29(0.2-0.37)	36.5(25.65-46.69)
	Vanuatu	0.38(0.19-0.55)	21.24(10.9-30.8)	0.05(0.02-0.07)	3.32(1.71-4.7)	1.13(0.56-1.63)	66.51(33.36-96.08)
South Asia	Bangladesh	91.52(69.89-146.08)	7.41(5.6-11.92)	11.43(8.56-19.09)	1.08(0.82-1.82)	234.06(175.18-380.07)	19.33(14.48-31.65)
	Bhutan	0.61(0.46-1.01)	9.98(7.48-16.1)	0.08(0.06-0.13)	1.57(1.17-2.51)	1.6(1.19-2.59)	27.11(20.27-43.88)
	India	985.35(919.54-1137.11)	9.03(8.42-10.4)	127.89(119.07-146.63)	1.4(1.3-1.61)	2675.28(2482.75-3094.86)	25.14(23.31-29)
	Nepal	16.9(13.01-25.78)	7.97(6.2-11.97)	2.71(2.08-4)	1.48(1.14-2.17)	53.91(41.57-81.15)	25.76(19.9-38.36)
	Pakistan	339.34(259.42-417.69)	29.86(23.23-36.39)	46.59(35.89-56.94)	5.2(4.12-6.23)	1056.86(804-1297.42)	96.65(74.2-118.28)
Southeast	Cambodia	20(15.35-24.05)	17.26(13.17-20.76)	2.39(1.79-2.9)	2.55(1.89-3.1)	51.91(39.39-62.6)	47.11(35.53-56.89)

Asia	Indonesia	451.5(318.67-551.72)	20.58(14.63-25.06)	46.34(32.89-56.61)	2.69(1.93-3.28)	1001.59(701.62-1226.69)	48.83(34.48-59.64)
	Laos	7.63(5.36-9.96)	17.52(12.19-22.79)	0.92(0.63-1.2)	2.65(1.83-3.42)	20.51(14.15-26.92)	49.98(34.28-65.19)
	Malaysia	71.65(61.22-88.61)	27.64(23.83-33.77)	5.2(4.45-6.31)	2.53(2.13-3.06)	101.03(86.33-123.89)	42.14(36.01-51.41)
	Maldives	0.94(0.81-1.1)	31.44(27.27-37.14)	0.06(0.05-0.07)	2.39(2.09-2.89)	1.13(0.99-1.34)	41.23(35.68-49.19)
	Mauritius	4.73(4.27-5.24)	27.39(24.76-30.26)	0.29(0.26-0.32)	1.88(1.7-2.06)	6.17(5.57-6.8)	36.79(33.38-40.49)
	Myanmar	97.27(77.94-115.09)	21.16(16.95-25.07)	11.56(9.11-13.96)	2.98(2.35-3.6)	243.12(192.83-291.24)	55.28(43.96-66.24)
	Philippines	85.05(73.49-98.31)	10.76(9.37-12.31)	6.51(5.72-7.39)	1.1(0.97-1.24)	155.77(135.11-178.12)	21.42(18.71-24.33)
	Seychelles	0.67(0.59-0.8)	59.68(53.31-71.55)	0.04(0.04-0.05)	4.58(4.12-5.56)	0.93(0.83-1.13)	87.24(77.73-106.27)
	Sri Lanka	45.88(35.27-55.79)	18.12(13.95-21.82)	2.87(2.22-3.43)	1.3(1.01-1.54)	58.44(44.57-70.85)	23.88(18.35-28.78)
	Thailand	228.23(196.41-290)	23.32(20.14-29.56)	14.87(13.15-18.09)	1.58(1.4-1.92)	289.52(251.55-364.89)	29.77(25.92-37.42)
	Timor-Leste	1.55(0.93-2.1)	19.23(11.89-25.81)	0.19(0.12-0.25)	2.68(1.75-3.51)	3.87(2.34-5.22)	48.96(30.23-65.15)
	Vietnam	126.52(106.63-154.1)	13.46(11.4-16.25)	9.96(8.52-12.14)	1.21(1.03-1.48)	204.37(172.46-251.71)	22.9(19.46-28.14)
Southern Latin America	Argentina	183.87(162.23-208.55)	34.86(30.74-39.59)	16.56(14.75-18.62)	3(2.67-3.38)	295.45(259.64-335.84)	55.27(48.48-62.81)
	Chile	83.08(73.62-94.52)	35.69(31.68-40.56)	6.01(5.39-6.7)	2.58(2.31-2.88)	105(92.95-117.92)	45.1(39.93-50.69)
	Uruguay	27.22(24.14-30.71)	52.87(46.66-59.94)	2.53(2.25-2.83)	4.26(3.77-4.78)	41.83(37-47.26)	78.15(69.23-88.67)
Southern Sub-Saharan Africa	Botswana	2.4(2.06-2.81)	16.58(14.47-19.39)	0.26(0.22-0.3)	2.27(1.97-2.66)	5.54(4.82-6.51)	40.81(35.73-47.82)
	Lesotho	1.58(1.07-2.11)	12.66(8.59-16.79)	0.25(0.17-0.32)	2.38(1.64-3.09)	5.82(3.94-7.8)	47.87(32.57-63.67)
	Namibia	1.53(1.28-1.8)	10.65(9-12.49)	0.21(0.18-0.25)	1.65(1.41-1.92)	4.36(3.67-5.1)	31.17(26.25-36.34)
	South Africa	86.85(72.38-93.48)	19(15.77-20.45)	9.18(7.7-9.84)	2.32(1.95-2.48)	197.74(166.67-213.84)	44.6(37.42-48.16)
	Swaziland	0.99(0.74-1.27)	16.25(12.28-20.8)	0.12(0.09-0.16)	2.6(2-3.29)	3.01(2.26-3.89)	51.83(39.43-66.89)
	Zimbabwe	23.88(16.44-29.89)	33.73(23.17-41.76)	3.55(2.43-4.39)	6.3(4.31-7.73)	86.78(58.7-108.26)	125.62(85.73-155.98)
Tropical Latin America	Brazil	519.05(504.61-535.89)	22.67(22.04-23.4)	48.82(47.44-50.39)	2.26(2.2-2.33)	917.47(887.32-949.7)	40.71(39.38-42.14)
	Paraguay	6.27(5.08-8.13)	11.65(9.43-15.38)	0.68(0.55-0.96)	1.39(1.12-1.98)	13.25(10.64-17.76)	25.32(20.3-34.52)
Western Europe	Andorra	1.24(0.94-1.49)	94.23(71.59-113.68)	0.07(0.05-0.08)	4.51(3.51-5.38)	1.05(0.8-1.26)	78.84(59.96-94.88)
	Austria	122.75(112.23-133.86)	72.59(66.33-79.31)	6.53(6.06-7.1)	3.24(2.99-3.52)	100.1(91.81-109.77)	56.2(51.48-61.87)

	Belgium	186.1(169.1-203.56)	86.51(78.34-94.89)	11.33(10.39-12.3)	4.34(3.98-4.71)	172.28(157.47-188.78)	75.84(69.11-83.05)
	Cyprus	15.15(10.94-17.75)	79.1(57.42-93.11)	0.8(0.6-0.93)	4.06(3.07-4.72)	14.24(10.37-16.66)	73.73(54.1-86.18)
	Denmark	111.4(102.7-121.5)	99.57(91.6-108.52)	6.3(5.83-6.8)	5.16(4.77-5.56)	103.19(94.75-112.16)	90.2(82.85-98.06)
	Finland	55.22(49.98-61.38)	46.97(42.24-52.18)	3.1(2.86-3.39)	2.27(2.09-2.5)	47.42(42.87-52.59)	38.76(35.02-42.93)
	France	1105.69(1001.73-1224.32)	88.78(79.97-99.19)	67.36(61.84-73.29)	4.25(3.87-4.64)	1020.68(920.44-1127.46)	77.4(69.7-86.16)
	Germany	1126.45(1001.82-1260.06)	62.71(55.66-70.28)	77.14(69.2-85.56)	3.59(3.21-3.98)	1177.18(1050.98-1315.81)	62.92(56.12-70.46)
	Greece	218.75(198.6-241.7)	100.63(91.32-112.03)	14.12(12.91-15.39)	5.1(4.68-5.54)	215.77(195.67-237.44)	92.16(83.71-101.16)
	Iceland	4.04(3.65-4.46)	77.21(69.93-85.4)	0.21(0.19-0.23)	3.58(3.28-3.93)	3.32(3.01-3.67)	61.9(56.08-68.57)
	Ireland	43.91(39.35-48.8)	60.89(54.53-67.71)	2.4(2.19-2.63)	3.17(2.89-3.48)	38.68(34.69-42.9)	52.94(47.44-58.68)
	Israel	65.53(58.53-72.74)	59.21(52.89-65.9)	4.59(4.14-5.04)	3.77(3.4-4.15)	70.13(62.69-77.6)	62.09(55.52-68.8)
	Italy	1383.33(1258.1-1519.6)	102.81(93.12-113.64)	76.34(69.72-83.57)	4.43(4.05-4.83)	1107.52(1003.95-1221.49)	76.1(68.74-84.06)
	Luxembourg	8.06(6.99-9.44)	86.12(74.39-101.08)	0.42(0.37-0.49)	4.07(3.57-4.72)	6.83(5.9-7.96)	70.8(61.05-82.69)
	Malta	6.71(6.05-7.41)	75.96(68.91-82.97)	0.38(0.35-0.42)	4.09(3.74-4.46)	6.44(5.84-7.05)	71.41(64.99-77.96)
	Netherlands	319.03(293.6-345.7)	98.1(90.21-106.19)	16.84(15.75-17.98)	4.67(4.36-4.99)	271.66(251.03-294.28)	81.29(75.13-88.12)
	Norway	73.87(69.05-78.36)	80.55(75.32-85.58)	3.98(3.78-4.16)	3.86(3.66-4.05)	62.33(58.49-66.1)	66.13(62.02-70.29)
	Portugal	166.39(149.94-185.39)	77.46(69.37-86.82)	10.74(9.85-11.74)	3.94(3.62-4.3)	163.66(148.37-179.93)	69.57(62.67-76.98)
	Spain	951.38(867.69-1049.52)	107.48(97.75-118.63)	58.59(53.85-63.79)	5.25(4.84-5.73)	884.69(809.88-972.93)	93.6(85.55-103.14)
	Sweden	146.19(136.12-157.22)	72.18(67.51-77.78)	8.45(7.92-9.01)	3.58(3.36-3.81)	128.18(119-137.85)	60.73(56.43-65.26)
	Switzerland	90.67(81.28-100.18)	55.31(49.3-61.16)	5.8(5.29-6.35)	3.02(2.75-3.31)	87.64(78.71-96.67)	51.29(45.92-56.67)
	United Kingdom	634.68(619.67-651.22)	52.86(51.57-54.2)	62.45(61.2-64.07)	4.4(4.31-4.51)	900.47(874.66-928.85)	71.35(69.3-73.71)
Western Sub-Saharan Africa	Benin	6.46(5.07-8.23)	13.53(10.73-17.22)	1(0.79-1.27)	2.54(2.03-3.24)	22.1(17.16-28.2)	47.69(37.61-60.94)
	Burkina Faso	10.71(7.38-14.13)	11.84(8.13-15.71)	1.61(1.09-2.15)	2.26(1.56-2.98)	36.59(24.61-48.73)	41.98(28.3-55.91)
	Cameroon	19.47(14.62-24.82)	16.49(12.48-21.09)	2.86(2.16-3.69)	3.08(2.32-3.95)	66.38(49.56-85.18)	58.29(43.73-75.13)
	Cape Verde	1.1(0.94-1.27)	23.4(20.25-26.99)	0.1(0.09-0.12)	2.3(2.02-2.79)	2.08(1.81-2.4)	46.24(40.36-54.1)
	Chad	7.15(5.63-9.12)	13.17(10.49-16.85)	1.23(0.98-1.58)	2.74(2.22-3.47)	27.21(21.32-34.94)	50.88(40.17-65.26)

	Cote d'Ivoire	15.28(11.62-19.58)	14.99(11.43-19.15)	2.36(1.76-3.02)	2.91(2.22-3.68)	56.36(41.93-72.69)	56.43(42.32-72.62)
	Ghana	32.81(26.36-40.75)	18.57(14.79-24.33)	3.57(2.8-5.21)	2.73(2.14-4.11)	89.6(70.83-117.41)	54.64(43.08-77.38)
	Guinea	12.43(9.93-15.09)	23.24(18.61-28.18)	2.3(1.85-2.78)	4.83(3.9-5.85)	48.55(38.5-59.1)	91.8(73.4-111.76)
	Guinea-Bissau	1.02(0.79-1.4)	14.02(11.18-18.94)	0.16(0.13-0.22)	2.94(2.42-3.86)	4.02(3.12-5.55)	56.91(45.08-77.11)
	Liberia	2.52(2.02-3.13)	12.71(10.22-15.72)	0.38(0.3-0.47)	2.43(1.96-3)	8.49(6.76-10.66)	44.42(35.56-55.58)
	Mali	37.8(28.8-48.1)	45.79(35.14-58.04)	5.68(4.31-7.2)	8.28(6.36-10.32)	125.05(94.39-160.68)	154.79(117.2-197.82)
	Mauritania	3.9(2.92-5.05)	19.11(14.55-24.32)	0.53(0.41-0.67)	3.08(2.42-3.85)	11(8.34-14.04)	55.89(42.84-71.1)
	Niger	6.46(3.83-9.5)	8.8(5.23-12.99)	0.98(0.57-1.47)	1.74(1.05-2.58)	23.05(13.3-34.46)	32.05(18.69-47.88)
	Nigeria	56.57(41.37-78.4)	7.06(5.25-9.58)	8.23(6.13-11.12)	1.27(0.95-1.66)	160.7(116.92-223.86)	20.9(15.39-28.48)
	Sao Tome and Principe	0.37(0.29-0.47)	31.73(24.59-43.09)	0.04(0.03-0.06)	5.02(3.83-7.13)	0.99(0.77-1.35)	93.49(71.26-133.21)
	Senegal	12.05(9.21-15.17)	16.64(12.8-20.83)	1.97(1.5-2.47)	3.18(2.43-4)	41.99(31.96-52.74)	59.06(44.93-74.38)
	Sierra Leone	4.84(3.58-6.15)	13.62(10.09-17.21)	0.75(0.54-0.96)	2.6(1.9-3.32)	16.72(12.11-21.42)	48.65(35.39-61.92)
	The Gambia	0.94(0.77-1.12)	10.05(8.24-11.95)	0.15(0.12-0.18)	1.85(1.52-2.19)	3.15(2.57-3.79)	34.56(28.13-41.17)
	Togo	4.81(3.29-6.39)	13.26(9.03-17.48)	0.67(0.45-0.89)	2.42(1.63-3.24)	16.3(10.96-21.77)	46.67(31.59-61.99)

ASP, age-standardized prevalence; ASDR, age-standardized death rate; DALYs, disability-adjusted life years; ASR, age-standardized rate
